# Genome Sequence of *Cronobacter sakazakii* BAA-894 and Comparative Genomic Hybridization Analysis with Other *Cronobacter* Species

**DOI:** 10.1371/journal.pone.0009556

**Published:** 2010-03-08

**Authors:** Eva Kucerova, Sandra W. Clifton, Xiao-Qin Xia, Fred Long, Steffen Porwollik, Lucinda Fulton, Catrina Fronick, Patrick Minx, Kim Kyung, Wesley Warren, Robert Fulton, Dongyan Feng, Aye Wollam, Neha Shah, Veena Bhonagiri, William E. Nash, Kymberlie Hallsworth-Pepin, Richard K. Wilson, Michael McClelland, Stephen J. Forsythe

**Affiliations:** 1 School of Science and Technology, Nottingham Trent University, Nottingham, United Kingdom; 2 Vaccine Research Institute of San Diego, San Diego, California, United States of America; 3 Genome Center at Washington University, Washington University School of Medicine, St. Louis, Missouri, United States of America; University of Hyderabad, India

## Abstract

**Background:**

The genus *Cronobacter* (formerly called *Enterobacter sakazakii*) is composed of five species; *C. sakazakii*, *C. malonaticus*, *C. turicensis*, *C. muytjensii*, and *C. dublinensis*. The genus includes opportunistic human pathogens, and the first three species have been associated with neonatal infections. The most severe diseases are caused in neonates and include fatal necrotizing enterocolitis and meningitis. The genetic basis of the diversity within the genus is unknown, and few virulence traits have been identified.

**Methodology/Principal Findings:**

We report here the first sequence of a member of this genus, *C. sakazakii* strain BAA-894. The genome of *Cronobacter sakazakii* strain BAA-894 comprises a 4.4 Mb chromosome (57% GC content) and two plasmids; 31 kb (51% GC) and 131 kb (56% GC). The genome was used to construct a 387,000 probe oligonucleotide tiling DNA microarray covering the whole genome. Comparative genomic hybridization (CGH) was undertaken on five other *C. sakazakii* strains, and representatives of the four other *Cronobacter* species. Among 4,382 annotated genes inspected in this study, about 55% of genes were common to all *C. sakazakii* strains and 43% were common to all *Cronobacter* strains, with 10–17% absence of genes.

**Conclusions/Significance:**

CGH highlighted 15 clusters of genes in *C. sakazakii* BAA-894 that were divergent or absent in more than half of the tested strains; six of these are of probable prophage origin. Putative virulence factors were identified in these prophage and in other variable regions. A number of genes unique to *Cronobacter* species associated with neonatal infections (*C. sakazakii*, *C. malonaticus* and *C. turicensis*) were identified. These included a copper and silver resistance system known to be linked to invasion of the blood-brain barrier by neonatal meningitic strains of *Escherichia coli*. In addition, genes encoding for multidrug efflux pumps and adhesins were identified that were unique to *C. sakazakii* strains from outbreaks in neonatal intensive care units.

## Introduction

*Cronobacter* spp. (formerly *Enterobacter sakazakii*) are Gram-negative, motile, non-sporeforming, peritrichous rods of the *Enterobacteriaceae* family. *Cronobacter* is a ubiquitous organism present in a wide range of environments, including water, soil, and a variety of processed foods and fresh produce [1]. The bacterium has been isolated from factory production lines including powdered infant formula factories and households [2] as well as from a wide range of clinical samples including cerebrospinal fluid, blood, bone marrow, sputum, urine and faeces [3]. The organism is an opportunistic pathogen of humans that can cause infections in all age groups. However, low birth weight neonates are most at risk. In this host group *Cronobacter* has been associated with outbreaks of necrotizing enterocolitis, meningitis and septicaemia. Infections with these presentations result in exceptionally high mortality rates ranging from 40 to 80 percent [4]. In recent years, some outbreaks of bacterial infection in neonatal intensive care units (NICU) have been traced to powdered formula contaminated with *Cronobacter* [5]–[8].

*Cronobacter* was defined as ‘yellow-pigmented *Enterobacter cloacae*’ until 1980, when it was designated a new species, *Enterobacter sakazakii*, by Farmer *et al* [9]. Analysis of both partial 16S rDNA and *hsp60* sequences showed that *E. sakazakii* isolates formed at least four distinct clusters, and it was proposed that clusters 2, 3, and 4 could be unique species [10]. Based on DNA-DNA hybridization and phenotyping, *Enterobacter sakazakii* was subsequently proposed to be re-classified into a new genus *Cronobacter*, composed of five distinct species: *Cronobacter sakazakii*, *C. malonaticus, C. turicensis*, *C. muytjensii* and *C. dublinensis* [11]. Due to their close relatedness *C. sakazakii* and *C. malonaticus* are difficult to distinguish by 16S rDNA sequence analysis. However, multilocus sequence typing (MLST) differentiates between the two species, and also reveals a strong clonal nature of the organism [12]. Previous studies on ‘*E. sakazakii*’ will therefore be difficult to interpret unless the strains are re-examined and re-classified according to the current taxonomic structure.

*Cronobacter* strains vary in their virulence, as determined by epidemiological studies and in-house mammalian tissue culture [6], [13], [14], but their virulence mechanisms are unknown. The bacteria can attach to intestinal cells and survive in macrophages [13], but the specific receptors involved remain to be determined. To date, only strains from *C. sakazakii*, *C. malonaticus* and *C. turicensis* have been associated with neonatal infections. Recently it was shown that the disruption of tight junctions significantly enhances association of *C. sakazakii* with Caco2 cells [15]. Some reports suggest a similarity between the tropism of *Cronobacter* and *Citrobacter koseri* for invasion and infection of the central nervous system [16], [17]. It was noted that brain abscesses due to *Cronobacter* and *Citrobacter koseri* were morphologically similar and may be due to similar virulence mechanisms [18]. The first putative *Cronobacter* virulence factors were enterotoxin-like compounds produced by four of eighteen strains [19]. The genes encoding the putative toxin have yet to be identified, however.

Here, we present the genome sequence of *C. sakazakii* strain BAA-894, isolated from powdered formula associated with a NICU outbreak [7], and use that sequence for comparative genomic hybridization (CGH) analysis of physiological and virulence related traits across the *Cronobacter* genus. Due to the severity of infant infection, a better understanding of the genomic variation between *Cronobacter* spp. is needed, and will be of interest to manufacturers of powdered infant formula, regulatory bodies, as well as those studying the evolution and diversity of pathogenicity.

## Results and Discussion

### *Cronobacter sakazakii* BAA-894 Genome

The complete sequencing of the genome of *C. sakazakii* BAA-894 revealed that it was composed of 1 chromosome (4.36837 Mb, 57% GC) and 2 plasmids (pESAK2 31 kb, 51% GC, pESAK3 131 kb, 56% GC); (Genbank accessions CP000783-5). A largely automated annotation of the genome resulted in the identification of 4,392 genes, covering 87% of the chromosome, 38 genes covering 83% of pESAK2 and 127 genes, covering 87% of pESAK3. The genome is aligned to many other enterobacterial genomes in the Enterix [20], [21] server (http://enterix.cbcb.umd.edu/enteric/enteric.html).

### Genome Cluster Analysis

In order to compare closely related genomes to the sequence of *C. sakazakii* BAA-894, we designed a set of 384,030 50-mer oligonucleotides that tiled the whole genome in both strands at an average density of about one oligonucleotide every 12 bases. An array was then manufactured by Roche NimbleGen (www.nimblegen.com).

Genomic diversity of 10 strains of *Cronobacter* representing the five different recognized species of this genus (Table 1) was analyzed by CGH on this tiled DNA microarray against the sequenced strain *C. sakazakii* BAA-894. *Cronobacter* genes were classified as present, absent or of intermediate status, as defined in the Materials and Methods section. The raw data is deposited in GenBank GEO, accession number GSE19308.

**Table 1:** Bacterial strains used in this study.

Organism	Organism ID	Comment	Source	% 16S rDNA sequence difference from *C. sakazakii* BAA-894	MLST sequence type	Reference
*C. sakazakii strain* BAA-894	Csak658	Genome sequenced strain.	Powdered formula	-	1	[7], [12]
*C. sakazakii* ATCC 29544^T^	Csak1	Species type strain.	Child's throat	0.38	8	[11], [12]
*C. sakazakii* ATCC 12868	Csak2		Unknown	0.57	3	[12]
*C. sakazakii* strain 20	Csak20		Clinical	0.57	4	[12]
*C. sakazakii* strain 701	Csak701	Fatal NEC III case	Peritoneal fluid	0.47	4	[6], [12]
*C. sakazakii* strain 767	Csak767	Fatal meningitis case	Trachea	0.47	4	[6], [12]
*C. sakazakii* strain 696	Csak696	NEC II case	Stools	0.57	12	[6], [12]
*C. malonaticus* LMG 23826^T^	Cmal1212	Species type strain.	Breast abscess isolate	0.57	NA	[11]
*C. turicensis* LMG 23827^T^	Ctur1211	Species type strain. Meningitis case	Blood isolate	1.80	NA	[11]
*C. muytjensiii* ATCC 51329^T^	Cmuy3	Species type strain.	Unknown	2.94	NA	[11]
*C. dublinensis* LMG 23823^T^	Cdub1210	Species type strain.	Environmental. Milk powder production plant	3.69	NA	[11]

**Figure 1:**
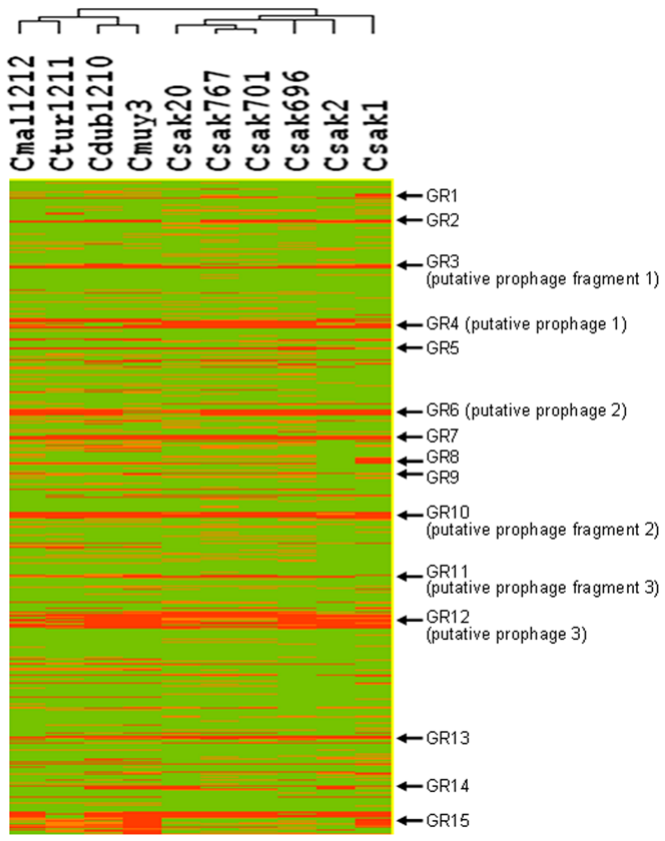
Whole genome clustering analysis showing the relationship between 10 *Cronobacter* isolates. Genomic regions GR1–GR15 are marked. Clustering analysis was performed using Gene Cluster (EisenSoftware). Hierarchical clustering was performed using the average linkage method on the trinary matrix based on the CGH analysis (1 for presence, 0 for uncertain and −1 for absence/divergence of a gene). For description of strains refer to Table 1.

To determine the presence or absence of genes, the median log_2_ ratio of the genome relative to the reference strain for all the oligonucleotides in that gene was calculated. Then GACK analysis [22] was used, which sets a floating threshold for presence and absence of genes for every hybridization (see below). Using Cluster and Treeview softwares [23], *Cronobacter* strains formed two distinct phylogenetic clusters. All *C. sakazakii* strains formed one cluster (Figure 1). *C. malonaticus, C. turicensis, C. dublinensis* and *C. malonaticus* formed a second, separate cluster. Within *C. sakazakii*, strains 701 and 767 were the most closely related and clustered together with strain 20. Previously, strains 701 and 767 were shown to belong to the same pulse field gel electrophoresis restriction digestion type [6]. Although the clinical details of the source of *C. sakazakii* strain 20 are unknown, the strain belongs to MLST sequence type 4 (as do 701 and 767), which is a stable clone of *C. sakazakii* isolated from both powdered infant formula and clinical sources [12]. *C. sakazakii* strain ATCC 29544^T^ (species type strain) formed a separate branch within the *C. sakazakii* cluster. The remaining *Cronobacter* species formed sub-clusters: *C. malonaticus* clustered with *C. turicensis* and *C. dublinensis* grouped with *C. muytjensii*. The tree remained identical when adjacent genes were collapsed into a single phylogenetic character if they had the same pattern of presence and absence.

### Core Genome

Of the 4,382 unique annotated gene sequences represented on the microarray, 54.9% (2404) were common to all *C. sakazakii* strains and 43.3% (1899) were common to all five *Cronobacter* species. The vast majority of these shared genes are predicted to encode cellular essential functions such as energy metabolism, biosynthesis, DNA, RNA and protein synthesis, cell division and membrane transport. The proportion of genes absent from test strains compared with *C. sakazakii* BAA-894 ranged from 10.3% (453) in *C. sakazakii* strain 20 to 17.1% (751) in *C. muytjensii* (Table 2). In total, 5.1% (224) of BAA-894 genes were absent in all *C. sakazakii* strains, and 3.1% (137) genes were absent in all *Cronobacter* strains (Table 2). Even though *C. muytjensii* and *C. malonaticus* are classified as separate species, the proportion of absent genes was only 11.3% and 11.9%, respectively, when compared to *C. sakazakii* BAA-894. This is in concordance with the previous 16S sequence comparison studies which showed that all *Cronobacter* strains are closely related (Table 1).

**Table 2:** Number of absent or divergent and intermediate genes in *Cronobacter* strains when compared to the sequenced *C. sakazakii* BAA-894.

Strain	Absent genes (%)^1^	Intermediate genes (%)^1^
*C. sakazakii* ATCC 29544^T^	582 (13.3)	449 (10.2)
*C. sakazakii* ATCC 12868	461 (10.5)	314 (7.2)
*C. sakazakii* strain 20	453 (10.3)	580 (13.2)
*C. sakazakii* strain 701	521 (11.9)	569 (13.0)
*C. sakazakii* strain 767	546 (12.5)	649 (14.8)
*C. sakazakii* strain 696	497 (11.3)	593 (13.5)
*C. malonaticus* LMG 23826^T^	522 (11.9)	608 (13.9)
*C. turicensis* LMG 23827^T^	495 (11.3)	591 (13.5)
*C. muytjensii* ATCC 51329^T^	751 (17.1)	522 (11.9)
*C. dublinensis* LMG 23823^T^	682 (15.6)	549 (12.5)
Absent in at least one *C. sakazakii*	835 (19.1)	
Absent in at least one *Cronobacter*	1081 (24.7)	

1 The numbers in brackets indicate the percentage of absent genes out of 4,382 annotated genes analyzed by comparative genomic hybridization.

### *C. sakazakii* Unique Genes

CGH analysis showed there were 21 genes present in all five *C. sakazakii* strains which were absent in *C. malonaticus, C. muytjensii, C. turicensis* and *C. dublinensis*. The genes unique to *C. sakazakii* strains were in two separate clusters of proteins involved in pilus assembly (ESA_02540–ESA_02542 and ESA_02796–ESA_02799), pilin FimA proteins (ESA_02541, ESA_02542, ESA_02796 and ESA_02799), porin PapC (ESA_02797) and the chaperone PapD (ESA_02798). The genes unique to *C. sakazakii* also included proteins for the phosphotransferase system (ESA_03303 and ESA_03305), a putative sialic acid transporter (ESA_03611), N-acetylneuraminate lyase (ESA_03612) and RelB from a toxin/antitoxin system (ESA_00257).

### Invasion of Brain Microvascular Endothelial Cells

Because *Cronobacter* is associated with often fatal cases of neonatal meningitis, the status of genes identified in other organisms as associated with invasion of brain microvascular endothelial cells (BMEC) (*ibeA, ibeB, yijP* and *ompA*) in the sequenced isolate was of particular interest [24]–[27]. The gene encoding OmpA was present in all tested strains. This protein is associated with the invasive ability of neonatal meningitic *E. coli*. While genes *ibeA* and *yijP* produced no match in the reference strain *C. sakazakii* BAA-894, *ibeB* (synonymous to *cusC*) was found. *CusC* belongs to a cluster of genes encoding a copper and silver resistance cation efflux system which allows bacteria to invade BMEC [28]. The complete cation efflux system c*usA* (ESA_04242), *cusB* (ESA_04241), *cusC* (ESA_04239), *cusF* (ESA_04240) and its regulatory gene *cusR* (ESA_04238) was present in strains associated with neonatal infections (*C. sakazakii* ATCC 29544^T^, 701, 767, 696, *C. turicensis* and *C. malonaticus*), and absent in the other tested strains.

### Other Physiological Traits

The presence of genes conferring physiological traits commonly associated with *Cronobacter* spp. was examined. Seventy genes involved in desiccation resistance [29], the metalloprotease *zpx* which causes rounding of Chinese hamster ovary (CHO) cells [30] and yellow pigment production genes [31] were present on the arrays. All these genes were present in all 10 *Cronobacter* strains tested.

### Comparison of *C. sakazakii* Neonatal Intensive Care Unit (NICU) Outbreak Strains with *C. sakazakii* Type Strain ATCC 29544^T^

The genes that were shared by the three strains associated with *C. sakazakii* outbreaks in NICUs (BAA-894, 701 & 767) were compared with the *C. sakazakii* species type strain ATCC 29544^T^, which showed decreased virulence properties compared to strains 701 and 767 in tissue culture studies [14]. One hundred and forty-four genes present in the three NICU strains were absent in the type strain, 66 (46%) in clusters of consecutive genes based on the annotation of BAA-894. In most of these clusters, genes encoding proteins associated with resistance to different forms of stress were identified, including multidrug efflux systems, genes involved in resistance to oxidative stress, and those with a putative function in resistance to metals. The complete list of genes present in NICU outbreak strains *C. sakazakii* BAA-894, 707 and 767 and absent in the *C. sakazakii* type strain ATCC 29544^T^ is in Table S1; genes of interest are listed below.

#### Category I

Genes encoding proteins associated with resistance to different forms of antibiotics: (i), a transcriptional regulator (ESA_01938) from the TetR family of protein repressors that control the level of susceptibility to hydrophobic antibiotics and detergents; (ii), a homologue of the CpmG protein involved in carbapenem resistance (ESA_pESA3p05435); (iii), a protein conferring resistance to antimicrobial peptides Mig-14 (ESA_pESA3p05439); and (iv), a transcriptional regulator (ESA_pESA3p05448) involved in tetracycline resistance.

#### Category II

Genes encoding multidrug efflux systems: (i), a cationic drug transporter (ESA_01940) from the family of proteins that confer resistance to a wide range of toxic compounds; (ii), genes for complete bacterial ABC-transport systems involved in active transport across the cytoplasmic membrane (ESA_01944–ESA_01946); and (iii), a variety of genes encoding multidrug efflux components located on the plasmid pESA3 in BAA-894 (Table S1).

#### Category III

Genes involved in resistance to oxidative stress and genes with a putative function in resistance to metals: (i), a redox-sensitive transcriptional activator SoxR (ESA_00115); (ii), a glutathione S-transferase (ESA_00116); (iii), ADP-ribose pyrophosphatase involved in oxidative stress protection (ESA_pESA3p05446); (iv), an arsenate reductase (ESA_pESA3p05485); and (v), a predicted transcriptional regulator involved in mercurium resistance ESA_pESA3p05463.

#### Category IV

Other genes of interest include: (i), putative adhesins which are recognized as virulence factors in enteric bacteria [32] (ESA_00983–ESA_00986); (ii), the universal stress protein UspA (ESA_01955) which can enhance the rate of cell survival during prolonged exposure to stress conditions [33]; (iii), a gene encoding a Type VI secretion lysozyme-related protein (ESA_02735); (iv), a gene for a predicted virulence SciE-type protein (ESA_02736) which affects the ability of bacteria to enter eukaryotic cells [34]; and (v), genes involved in pilus assembly (ESA_03515 and ESA_03516).

### *C. sakazakii* Plasmids

The sequenced strain *C. sakazakii* BAA-894 contains two plasmids; pESA2 (31 kb) and pESA3 (131 kb). Thirty-eight genes were annotated on pESA2 and 127 genes on pESA3.

The copy number of the plasmids was estimated from the median hybridization signals of oligonucleotides representing the plasmid compared to the sequenced genome. The ratio was (1∶1.1∶8.6) for the chromosome versus pESA2 versus pESA3. Thus, pESA2 exists as low copy, and pESA3 appears to be a moderate copy number plasmid.

The genes on pESA2 were absent in all other strains tested except *C. turicensis*, which had 19 (61.3%) genes present, and *C. sakazakii* 696, which had 4 (12.9%) genes present. The results for genes on pESA3 are summarized in Table 3.

**Table 3:** pESA3 genes present in *Cronobacter* strains when compared to the sequenced strain of *C. sakazakii* BAA-894.

Strain	Present genes (%) 1	Plasmid profile 2
*C. sakazakii* ATCC 12868	103 (88.8)	+
*C. sakazakii* 696	81 (69.8)	+
*C. sakazakii* 767	77 (66.4)	+
*C. sakazakii* 20	73 (62.9)	+
*C. sakazakii* 701	61 (52.6)	+
*C. malonaticus* LMG 23826^T^	57 (49.1)	+ (3)
*C. sakazakii* ATCC 29544^T^	51 (44.0)	+ (3)
*C. turicensis* LMG 23827^T^	35 (30.2)	−
*C. dublinensis* LMG 23823^T^	19 (16.4)	−
*C. muytjensii* ATCC 51329^T^	0 (0.0)	−

1 Of the 127 annotated genes on pESA3, 116 were analyzed by comparative genomic hybridization. The other genes did not have a sufficient number of probes that passed the filters for manufacture on the array.

2 Plasmid of a size similar to pESA3 (131 kb) was detected by gel electophoresis of plasmid isolations.

3 The detected plasmid was smaller in size than pESA3 (110 kb).

Note that it is possible that some or all of the detected genes are on the chromosome in other strains. In addition, genes on a multicopy or medium copy plasmid may require a different degree of divergence to be identified as absent or divergent by comparative hybridizations. Plasmid profiling was performed on the Cronobacter strains analyzed by comparative hybridization,. A plasmid of a size similar to pESA2 (31 kb) was detected in *C. sakazakii* 696 and in *C. turicensis*, which is in accordance with our CGH results. A large plasmid similar in size to pESA3 (131 kb) was visible in *C. sakazakii* strains ATCC 29544^T^, ATCC 2868, 20, 696, 701, 767 and *C. malonaticus* (Table 3).

### Genomic Regions Absent in Some Strains of *Cronobacter*

**Figure 2:**
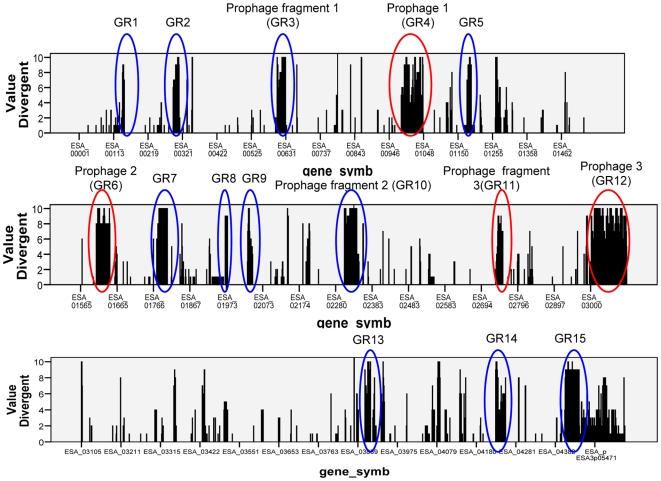
Distribution of the 15 variable regions across the *C. sakazakii* BAA-894 genome. Each column represents a gene classified as absent by CGH analysis in at least one strain. The height of the columns indicate the number of strains (out of 10) in which the gene was found to be absent. The major variable regions (blue) and prophages (red) are indicated in order of their appearance in the genome of *C. sakazakii* BAA-894.

Genes that were absent in more than half of the *Cronobacter* strains relative to the sequenced strain *C. sakazakii* BAA-894 were selected for further analysis. These genes form 15 clusters of contiguous genes (based on the annotation of the reference genome). These are shown on Figure 2, where the number of strains in which a particular gene was classified as absent is plotted against the gene locus. The clusters were designated as regions GR1–GR15.

### Prophages

Of the 15 clusters, three putative prophage genomes and one prophage fragment were identified by Prophinder [35], and two additional regions are probable prophage fragments based on the presence of phage protein homologues identified by BLASTX (Table 3).

In the three prophage gene clusters (prophages 1, 2 and 3), genes encoding close homologues of known phage genes involved in integration, lysis and termination as well as head and tail structure were identified based on amino acid identity searches in IMG-JGI (http://img.jgi.doe.gov/cgi-bin/pub/main.cgi). The average GC content of the sequenced *C. sakazakii* BAA-894 genome is 56%, the GC content of prophages 1, 2 and 3 was 53, 49 and 51%, respectively. The complete list of annotated putative prophage genes is available Table S2. In addition, Figure S1. shows the status of all putative prophage genes in the 10 *Cronobacter* strains.

**Figure 3:**
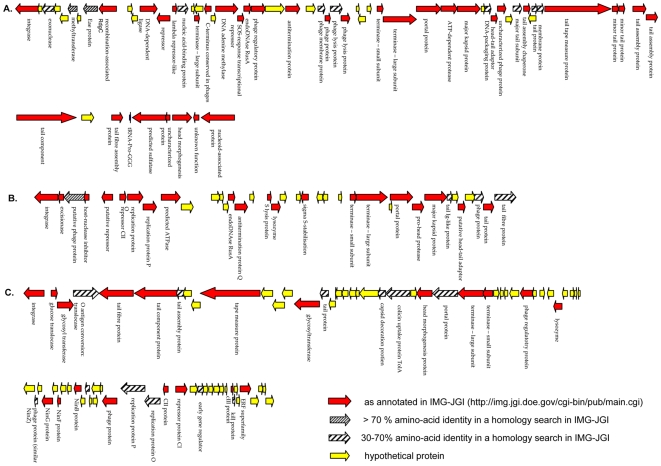
Gene maps of putative prophages. A. Gene map of putative prophage 1. B. Gene map of putative prophage 2. C. Gene map of putative prophage 3. Annotation of the putative prophage genes is available in Table S2.

**Prophage 1** (**GR4**; ESA_00990–ESA_01052). In the 46-kb putative prophage 1 (Figure 3A), 30/63 (48%) hypothetical proteins were similar to known phage proteins. Homologues of phage genes involved in integration, lysis, head morphogenesis, tail assembly and phage regulation were identified. Prophage 1 also contains a gene (ESA_00997) encoding a protein homologous to the *eae*-like adhesion protein associated with the attaching and effacing phenotype. The *eae* gene is carried by some other bacteriophages of enteric pathogens: *Salmonella* phage epsilon34, *E. coli* O157∶H7 bacteriophage PhiV10, enterobacterial phage P22 and enterobacterial phage epsilon15.

**Prophage 2** (**GR6**; ESA_01608–ESA_01644). Putative prophage 2 (Figure 3B) contains 37 genes, out of which 25 (68%) were homologous to known phage proteins. Prophage 2 contains several lambdoid phage genes encoding the following proteins: repressor CII (ESA_01613), replication proteins O and P (ESA_01614 and ESA_01615), the antitermination protein Q (ESA_01622), small and large subunits of the phage terminase (ESA_01632 and ESA_01633) as well as head and tail morphogenesis proteins. Head morphogenesis genes (ESA_01635–ESA_01637) were similar to head proteins of bacteriophage HK97 from the family of lambda phages. Two gene clusters have very low average GC content; 33% (ESA_01616–ESA_01620) and 44% (ESA_01627–ESA_01631). Both these clusters contain hypothetical proteins that showed no similarity with known phage proteins or functions.

**Prophage 3** (**GR12**; ESA_03025–ESA_03102) was the largest (47 kb) putative prophage identified (Figure 3C). Thirty-four genes (39%) genes had close homology to known phage proteins or functions. The rest of the annotated genes are conserved proteins of unknown functions or hypothetical proteins. Similarly to prophage 2, several regulatory genes characteristic for lambdoid phages were identified: repressor proteins CI, CII and CIII, early gene regulator protein, replication proteins O and P as well as N independence proteins NinBFGZ. A cluster of three O-antigen conversion genes (ESA_03026–ESA_03028) was found in putative prophage 3 between phage integrase and tail morphogenesis genes (Figure 3C). The putative colicin uptake protein TolA (ESA_03048) may be involved in the internalization of the bacteriophage, as the Tol pathway can be also used for the translocation of phages into the bacterial cell [36]. CGH showed that the entire genome of prophage 3 or its close relatives are absent from the genomes of all other *Cronobacter* strains tested except *C. turicensis*. In this species, 18 prophage genes (mostly annotated as hypothetical proteins) were classified as present (Figure S1). Interestingly, the CIII regulator protein (ESA_03094), the Kil protein (ESA_03095), and both large and small terminase subunits (ESA_03052 and ESA_03053) were present in two *C. sakazakii* strains (701 and 767), possibly as a part of different bacteriophages. These two strains were isolated from two fatal neonate cases of *C. sakazakii* infection [6]. A cluster of putative phage tail proteins (ESA_03029–ESA_03034) and a cluster containing phage head morphogenesis genes and a putative colicin uptake gene (ESA_03039–ESA_03051) were homologous to genes of the *S. enterica* serovar Typhi Vi type II phage E1 which may use virulence-associated capsular antigen as entry. This antigen was present on the surface of clinical Typhi isolates [37]. Although most genes of the putative prophage 3 were absent in all 10 *Cronobacter* strains tested by CGH, the region corresponding to the phage Vi genes (ESA_03041–ESA_03048) was present in *C. turicensis* and partially present in *C. sakazakii* strains 20, 701 and 767 (Figure S1). Strains 701 and 767 were both associated with fatal outbreaks, and are in MLST sequence type 4 with strain 20 [12].

**Prophage fragment 1** (**GR3**; ESA_00604–ESA_00630). Eight of 19 genes in this region encode proteins associated with phages: plasmid and phage DNA primase (ESA_00620), a protein from Ash phage family (ESA_00624), the phage transcriptional regulator AlpA (ESA_00625), a putative phage capsid protein (ESA_00626), the phage transcriptional activator Ogr/Delta (ESA_00627) and phage integrase (ESA_00630). ESA_00618 was homologous to ea59 of lambda bacteriophage and ESA_00622 was homologous to a P4 phage protein. This cluster is most probably a phage remnant and may not encode a functional phage due to the absence of homologues of known structural tail proteins. The phage cluster was absent in all other strains. However, a short region, ESA_00609–ESA_00617, was present in *C. sakazakii* strains 701 and 767. A group of restriction endonucleases belonging to this cluster encoding a restriction-modification methyltransferase subunit (ESA_00614), a restriction endonuclease S subunit (ESA_00615), a hypothetical protein (ESA_00616) and a type I site-specific deoxyribonuclease (ESA_00617) were homologous to genes api49, api50, api51 and api52, respectively, from the *Yersinia pseudotuberculosis* adhesion pathogenicity island [38].

**Prophage fragment 2** (**GR10**). The region ESA_02304–ESA_02339 is likely to represent another prophage remnant. The region mostly contains hypothetical proteins with unknown functions. However, eight genes showed some degree of homology to proteins of phage origin. The cluster is flanked by a gene homologous to phage methyltransferase (ESA_02304) and a gene containing a site-specific recombinase domain that is found in putative integrases/recombinases of mobile genetic elements of diverse bacteria and phages (ESA_02339). It also includes genes homologous to phage lysozyme (ESA_02309), a phage tail component (ESA_02311), a putative phage tape measure protein (ESA_02313), another unspecified phage protein (ESA_02316), a major capsid protein (ESA_02319) and a phage portal protein (ESA_02320). GR10 might be another remnant of a prophage that has previously integrated into *C. sakazakii* BAA-894 genome. The presence of putative integrase flanking the cluster suggests introduction of this cluster into the genome by horizontal gene transfer. This cluster was absent in all *Cronobacter* strains except the genes encoding phage lysozyme (ESA_02309) and a hypothetical protein (ESA_02310), present in *C. sakazakii* strains 2 and 20, probably as a part of different prophages.

**Prophage fragment 3** (**GR11**; ESA_02740–ESA_02755). The gene cluster of the 8 kb putative prophage fragment 3 comprises 16 hypothetical proteins, 10 of which (63%) may be associated with phage functions. As it lacks genes for head and tail morphogenesis as well as phage regulatory genes, it is likely to be a non-functional phage remnant.

Two thirds of all gamma-proteobacteria and low GC Gram-negative bacteria harbor prophages [39]. There is an increasing body of evidence that phages play a pivotal role in the diversification of bacterial species. Some phages can carry additional cargo genes, which are not required for the phage cycle and are suspected or proven virulence factors [39]. Such genes are typically located near prophage ends, downstream of phage tail genes or next to Q or N-like antiterminator genes [40]. Putative prophages identified in this study contain genes which are not similar to any other known prophage genes. Moreover, prophage 1 contains a gene encoding a protein homologous to the *eae*-like adhesion protein, which is a recognized virulence factor in enteropathogenic *E*. *coli* associated with the attaching and effacing phenotype [41]. It is hypothesized that most prophages are lost from bacterial genomes shortly after their acquisition [39]. Hence, some of the cargo genes carried by the prophages remaining in the chromosome of *Cronobacter* are possible virulence factors or fitness factors that increase the survival of the bacterium in its host. Further research into these putative virulence factors is warranted.

### Non-Phage Regions That Differ among the Strains

The complete list of genes belonging to the variable non-phage regions and their presence in tested strains is available in Table S2.

**GR1** (ESA_00140–ESA_00145) is a small cluster of type VI secretion system genes. ESA_00142 shares a conserved region with a family of IcmF-related proteins proposed to be involved in increased *Vibrio cholerae* adherence to epithelial cells [42]. ESA_00143 is a secretion protein belonging to the VC_A0110 family; mutations in proteins of this family are associated with impaired virulence [43]. ESA_00145, a secretion lipoprotein from a VC _A0113 family was present in *C. sakazakii* strains 701 and 767 associated with fatal outbreaks. The rest of GR1 was absent in all tested strains except *C. sakazakii* 696.

**GR2** (ESA_00292–ESA_00310) mostly contains uncharacterized conserved proteins. It also contains the gene encoding a protein from a family of beta-lactamases (ESA_00299).

**GR5** (ESA_01179–ESA_01189) contains a cluster of proteins involved in cell wall biogenesis and nucleotide sugar metabolism. GR5 corresponds to the *C. sakazakii* O-antigen gene locus used to distinguish the two *Cronobacter* serotypes O∶1 and O∶2 [44]. DNA microarray analysis revealed that GR5 is highly divergent; its genes were not sufficiently similar to be detected by microarray hybridization in any other *Cronobacter* strains. The O-antigen locus contains two more genes (homologues of ESA_01177 and ESA_01178) which were present in all strains except *C. sakazakii* 696.

**GR7** (ESA_01775–ESA_01804). Most genes in GR7 were predicted to be involved in tellurite and stress resistance. It contains homologues of tellurite resistance proteins TerA, TerC, TerD, TerY and TerZ. The cluster contains two putative transposase genes, which suggests that the cluster was acquired by horizontal transfer. GR7 was found to be carried on plasmid pK29 of *Klebsiella pneumoniae* strain NK29, plasmid pEC-IMPQ of *Enterobacter cloacae*, plasmid R478 of *Serratia marcescens* and plasmid pAPEC-O1-R of *Escherichia coli* APEC O1, which is further evidence of horizontal gene transfer. As the gene cluster was entirely absent from all other *Cronobacter* strains in our study, the reference strain BAA-894 probably acquired the tellurite resistance cluster recently.

**GR8** (ESA_01970–ESA_01976) contains seven genes encoding pilus assembly proteins. Fimbriae (or pili) enable bacteria to colonize the epithelium of specific host organs and are therefore considered major virulence factors [45]. This cluster of genes was absent in all *Cronobacter* strains except *C. sakazakii* strain 20.

**GR9** (ESA_02032–ESA_02041) contains genes encoding hypothetical proteins and four proteins involved in Type VI secretion system (ESA_02037–ESA_02040). These four genes were present in *C. sakazakii* strains 1 and 20 and absent in all other strains.

**GR13** (ESA_03887–ESA_03912) is a cluster of 16 hypothetical proteins without homology to known proteins or functions. This cluster was absent in all other *Cronobacter* strains.

**GR14** (ESA_04248–ESA_04255) is a cluster of genes involved in copper resistance (Table 4). This cluster was found in *C. sakazakii* strain 1 and 696, as well as *C. turicensis* and *C. malonaticus*.

**Table 4:** Putative prophages identified in the genome of *C. sakazakii* BAA-894 by Prophinder [35] or BlastX.

Designation	Prophinder ID	Size (kb)	Coordinates
Prophage 1 (GR4)	48917	45.7	969835–1015482
Prophage 2 (GR6)	48912	26.7	1561010–1587672
Prophage 3 (GR12)	48916	46.9	2974743–3021625
Prophage fragment 1 (GR3)	48908	8.1	2693304–2701447
Prophage fragment 2 (GR10)	BlastX	33.4	2237406–2270858
Prophage fragment 3 (GR11)	BlastX	8.2	2693304–2701466

**GR15** (ESA_pESA3p05493–ESA_pESA3p05505) involves genes located on *C. sakazakii* plasmid pESA3 and includes components of type IV and type VI secretion pathways (Table S2) as well as a gene encoding an outer membrane protein from the OmpA family (ESA_pESA3p05495). GR15 was absent from all strains except *C. sakazakii* strains 1 and 696.

Most of the described regions contain suspected or proven virulence factors. The genes in GR1, GR9 and GR15 are involved in a type VI secretion system, a newly described mechanism for protein transport across the cell envelope of Gram-negative bacteria that can increase adherence to epithelial cells [46], [47]. GR3 contains four genes (ESA_00614–ESA_00617) that are homologous to a restriction-modification gene cluster (api49–api52) in the *Yersinia pseudotuberculosis* pathogenicity island (YAPI) [38]. As these genes were present in strains 701 and 767 isolated from two neonates that died as a result of infection by *Cronobacter* during an outbreak in France [8], [17], and are absent in all other strains tested by CGH, these genes may be important virulence factors contributing to the pathogenicity of *Cronobacter*.

*Cronobacter* virulence factors have not been extensively studied, although it is known that *Cronobacter* species vary in their virulence with respect to invasion of intestinal cells, survival in macrophages and serum resistance [13], [14]. GR 5 (ESA_01179–ESA_01189) encodes the lipopolysaccharide (LPS) genes. Characterisation of LPS structure and consequently O-antigen can be important in developing identification schemes based on serotyping, and has a role in virulence and serum resistance of the organism. The LPS is one of the few structural features of *Cronobacter* which has been investigated and it is known that it varies across the *Cronobacter* spp. In *C. sakazakii* and *C. malonaticus*, the LPS are composed of various branched polymers, whereas they are unbranched in *C. muytjensii*. In *C. sakazakii* BAA-894 [48] it is a branched polymer of pentasaccharide units composed of 2-acetamido-2-deoxy-D-galactose, 3-(N-acetyl-L-alanylamido)-3-deoxy-D-quinovose, D-glucuronic acid, and D-glucose. *C. sakazakii* strain 767 is also a branched polymer but of a repeating heptasaccharides composed of 2-acetamido-2-deoxy-D-glucose, and D-galacturonic acid, L-rhamnose, and D-glucose [49]. *C. malonaticus* LPS [50] is also a branched pentasaccharide unit of 2-amino-2-deoxy-D-glucose, 2-amino-2-deoxy-D-galactose, 3-deoxy-D-manno-oct-2-ulosonic acid, D-galactose and D-glucose residues. Whereas, *C. muytjensii* LPS [51] is a linear unbranched pentasccharide polymer of 2-acetamido-2-deoxy-D-galactose, 2-acetamido-2-deoxy-D-glucose, 2-acetamido-3-deoxy-D-quinovose, L-rhamnose and D-glucuronic acid. These considerable differences correspond with the lack of sequence conservation in GR5 as revealed in the microarray analysis. The individual genes encoding these differences in enyzmology have yet to be assigned.

### Comparison to Other Enterobacterial Genera

The sequenced genome *C. sakazakii* BAA-894 was compared to the genomes of *Citrobacter koseri* BAA-895, *Klebsiella oxytoca* VJSK009, *E. coli* K12 MG1655 and *Salmonella enterica* Typhimurium strain LT2, representing some of the most closely related genera to *Cronobacter.* Using a threshold of identity of >85% in a 100 base window, 334 genes were present in all *Cronobacter* but absent or diverged in the four members of other genera (manuscript in preparation). These genes included a cluster of type VI secretion genes (ESA_03943 - ESA_03948) which might be involved in virulence, and a putative palatinose operon (ESA_02709 - ESA_02715). Alpha-glucosidase activity, which has been linked to palatinose metabolism, is considered as one of the major biochemical traits that distinguish *Cronobacter* from other related *Enterobacteriaceae*.

### Summary and Conclusions

Using a whole-genome ∼384,000 oligonucleotide tiling microarray, we analyzed the genomic content of isolates representing all five *Cronobacter* species by CGH. A dynamic determination of cut-offs GACK [22] was used to minimize the number of incorrectly categorized genes. Among 6 strains of *C. sakazakii* 2,404 genes (54.9%) represented a core shared genome. Of these genes 1,899 (43.3%) were also in the core genome when compared to four other *Cronobacter* species. CGH highlighted a copper/silver resistance cluster associated with invasion of BMEC, which were unique to the three *Cronobacter* species associated with neonatal infections, as well as efflux pumps and adhesins unique to *C. sakazakii* strains from NICU outbreaks.

The main genetic features that distinguished the *Cronobacter* strains were putative prophages and several other gene clusters, a pattern of divergence typical among bacteria [52]. A few of the regions present in the sequenced strain and absent in some other *Cronobacter* strains are found in only a few other *Enterobacteriaceae*. For example, GR 7 is found in four of the hundreds of *Enterobacteriaceae* genomes that have been sequenced, which indicates that this region may have been horizontally acquired, possibly from a source outside of the *Enterobateriaeciae*. We have shown that gene acquisition via integration of phages and other mobile elements and specific gene-loss play a major role in *Cronobacter* evolution and diversity. Fifteen clusters of genes including three putative prophages and three putative prophage fragments that were absent in more than half tested strains were identified. In most of them, putative virulence genes were identified.

Future studies will focus on the expression of virulence related genes and their role in the pathogenicity of *Cronobacter* species, particularly the mechanisms of neonatal infection.

## Materials and Methods

### Strains and Culture Conditions

*Cronobacter* strains were selected which represented the five recognized species, and included those from reported clinical cases (Table 1). All *Cronobacter* strains were stored at −80°C in Nutrient Broth (Oxoid, UK) with 10% glycerol, subcultured on Trypticase Soy Agar (Oxoid, UK) and checked for purity. Overnight Trypticase Soy Broth (Oxoid, UK) cultures were used for DNA extraction.

### DNA Extraction

Total genomic DNA was isolated using the QIAGEN Genomic-tip 100/G and Genomic DNA Buffer Set (www.1.qiagen.com) with extended cell lysis (1 h) and two additional washes of the precipitated DNA. The DNA samples were checked for fragmentation on 0.8% agarose gels and checked for protein and RNA content by spectrophotometry.

### Sequencing of *C. sakazakii* ATCC BAA-894 and Assembly

The complete genome of *C. sakazakii* BAA-894, a strain isolated from a powdered formula used during an NICU outbreak [7], was sequenced using the whole genome shotgun method, supplemented with end sequencing of a fosmid library. Sonicated and size-fractionated DNA was cloned into plasmid vectors (pOTw13). Subclones and fosmids were sequenced using dye-primer and dye-terminator chemistry on ABI 3730 sequencing robots. Using the PCAP assembly software program [53], 51,289 sequence reads, representing 6.2 fold coverage, were assembled. Part of the 6.2X coverage included 1.19X fosmids.Under-represented areas, gaps, and ambiguities were then addressed by performing automated sequence improvement [54] using directed sequencing from the subclones (plasmids and fosmids). Following the auto-finish process, correcting misassembled regions, resolving ambiguous bases, and filling the remaining gaps by additional directed sequencing and PCR, completed the finishing process. This yielded a product with a final estimated accuracy of 99.99%.

### Annotation

AceDB was the primary annotation database. The identification of protein-coding genes used a combination of GeneMark, Glimmer 2.0 and Glimmer 3.0; an evidence-based approach was used to prioritize genes for inclusion into a final gene set. Genes missed by the two *ab initio* gene predictors were identified using BlastX.

### *C. sakazakii* ATCC BAA-894 Microarray Design and Comparative Genome Hybridization Analysis (CGH)

A 384,030 probe oligonucleotide tiling DNA microarray was designed which comprised the complete genomic sequence of *C. sakazakii* ATCC BAA-894. Probes were designed at an average of less than 12 base spacing on alternating strands, leading to an average of over 100 50-mer oligonucleotide probes per annotated gene.

Every possible 50-base probe from both strands of the ESA genome and two plasmids was tested for the ability to be manufactured by NimbleGen. Those that required too many NimbleGen cycles were shortened. Resulting candidate probes that were less than 35 bases long were thrown out. The remaining 9,061,350 potential probes had an average melting temperature (Tm) of 74 degrees Celcius. Probes that had Tm above the average were shorted, down to a minimum of 35 bases. The probes were selected from the pool of 9,061,350 potential probes by selecting the best probe at 11.375-base increments, alternating between strands each time. The resulting 386,802 candidate probe sequences where analyzed to remove any probes that covered the same region as another probe due to duplications in the genome and the resulting 384,030 unique sequences were chosen for the array. Mappings between the unique probe sequences and their genome/plasmid/gene positions were stored in a separate file.

Sample labeling, CGH and data normalization were performed according to the method described at http://www.nimblegen.com/products/lit/cgh_userguide_v5p1.pdf.

DNA from the sequenced strain, *C. sakazakii* BAA-894, was used as the internal array control. For within-array normalization, a LOWESS method [55] was used as spatial correction and QSPline [56] was used to correct for dye bias. The raw data is deposited in GenBank GEO (accession number GSE19308).

### Data Visualization by WebArrayDB

The CGH plotter available at WebArray (www.webarraydb.org/webarray/index.html) was used to create CGH plots. This used the log_2_ intensity ratio microarray data to calculate the median log_2_ intensity ratios for each *C. sakazakii* BAA-894 gene. WebArrayDB is a database system and online cross-platform analysis suite for analysis of microarray data, which allows storage of the data in the repository and their online analysis [57].

### Dynamic Cut-Off Determination by GACK

Each gene was represented by tens or hundreds of separate oligonucleotides. These measurmements were condensed into a single median ratio for each gene. The data were further analyzed using the dynamic cut-off determination tool GACK [22]. The normal probability density function was calculated from the characteristics of the main peak of the data distribution. This was used to calculate the estimated probability of presence that gives a statistical validation of the gene assignment. Each CGH experiment was attributed a specific set of cut-offs to minimize the number of falsely assigned genes.

*Cronobacter* genes were classified according to the most stringent settings of the trinary output of GACK as present, intermediate or absent. Genes classified as ‘present’ had a log_2_ intensity ratio (test/reference) greater than the cut-off value corresponding to the 100% estimated probability of presence (EPP) calculated by GACK. The ‘absent’ genes had a log_2_ ratio inferior to the cut-off value corresponding to 0% EPP, and can include genes with sufficient sequence divergence. The ‘intermediate’ category included genes whose status could not be assigned with certainty. The EPP function and cut-offs were determined separately for hybridization data of each strain (Table S3).

### Prophage Identification

ACLAME Prophinder was used for prophage identification [35]. This was accessed at http://aclame.ulb.ac.be/Tools/Prophinder/

### 16S rDNA Sequence Analysis

Partial 16S rDNA sequence analysis was done as previously described [10].

### Plasmid Profiling

Plasmid DNA was isolated according to the method described in [58].

## Supporting Information

Figure S1 Absence/presence status of prophage genes in *Cronobacter* strains. Red indicates absence/divergence of a particular gene, orange indicates uncertain status and green indicates presence of a gene.
(0.15 MB TIF)

Table S1 Selected genomic regions present in NICU outbreak strains *C. sakazakii* 707 and 767 and absent in *C. sakazakii* type strain ATCC 29544T.
(0.16 MB DOC)

Table S2 Non-phage genomic regions absent in more than half of the tested strains.
(0.40 MB DOC)

Table S3 Cut-off values used for the assignment of absent, intermediate or present gene status.
(0.04 MB DOC)
