# Supplementary figures and images for: Genome Sequence of Cronobacter sakazakii BAA-894 and Comparative Genomic Hybridization Analysis with Other Cronobacter Species
Source: PLoS One. 2010 Mar 8;5(3):e9556. doi: 10.1371/journal.pone.0009556 (PMC2833190; doi:10.1371/journal.pone.0009556)

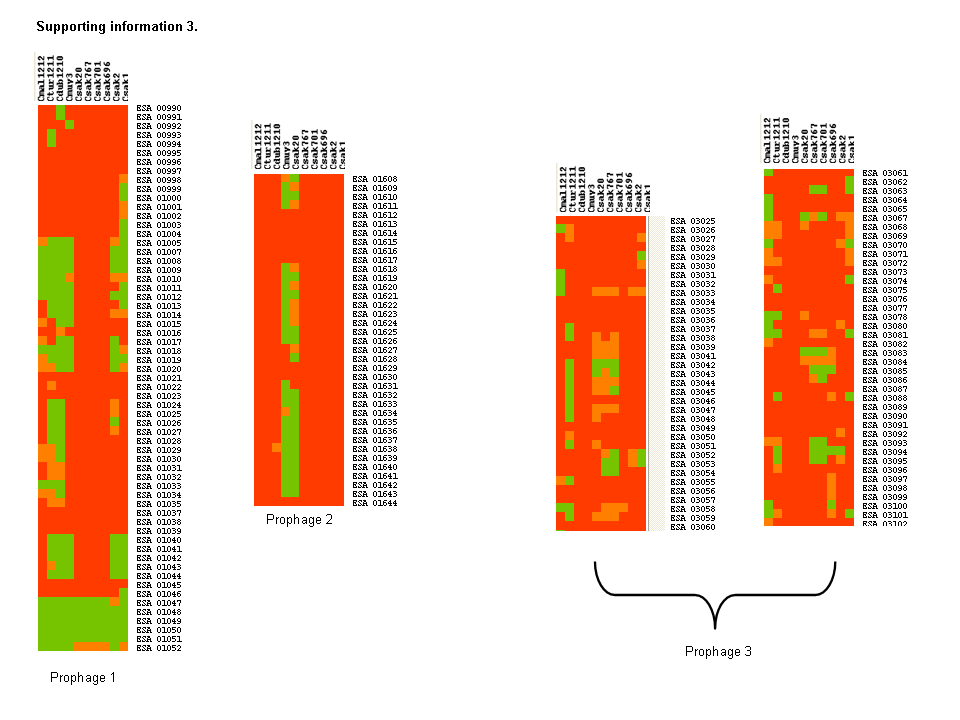

Supplement: Figure S1 — Absence/presence status of prophage genes in Cronobacter strains. Red indicates absence/divergence of a particular gene, orange indicates uncertain status and green indicates presence of a gene. (0.15 MB TIF) [file pone.0009556.s001.tif]
