# Supplementary material for: Genome Sequence of Cronobacter sakazakii BAA-894 and Comparative Genomic Hybridization Analysis with Other Cronobacter Species
Source: PLoS One. 2010 Mar 8;5(3):e9556. doi: 10.1371/journal.pone.0009556 (PMC2833190; doi:10.1371/journal.pone.0009556)
Supplement: Table S1 — Selected genomic regions present in NICU outbreak strains C. sakazakii 707 and 767 and absent in C. sakazakii type strain ATCC 29544T. (0.16 MB DOC) [file pone.0009556.s002.doc]

**Table S1.** Selected genomic regions present in NICU outbreak strains *C. sakazakii* 707 and 767 and absent in *C. sakazakii* type strain ATCC 29544^T^.

| Gene locus | Product description |
| --- | --- |
| Cluster 1 |  |
| ESA_00114 | Transcriptional regulator, AraC family |
| ESA_00115 | Redox-sensitive transcriptional activator SoxR, response to oxidative stress. |
| ESA_00116 | Glutathione S-transferase |
| ESA_00117 | Permeases |
| ESA_00118 | Na+/H+ antiporter, bacterial form |
| ESA_00120 | Cation/acetate symporter ActP |
| ESA_00122 | Acetyl-coenzyme A synthetase |
| ESA_00126 | Molecular chaperone, HSP90 family |
| ESA_00127 | Hypothetical protein |
| ESA_00128 | Hypothetical protein |
| ESA_00145 | Type VI secretion lipoprotein, VC_A0113 family |
| ESA_00313 | Hypothetical protein |
| ESA_00349 | Hypothetical protein |
| ESA_00562 | Amino acid ABC transporter substrate-binding protein, PAAT family (TC 3.A.1.3.-) |
| ESA_00605 | Hypothetical protein |
| Cluster 2 |  |
| ESA_00609 | Hypothetical protein |
| ESA_00610 | Superfamily II DNA/RNA helicases, SNF2 family |
| ESA_00611 | Hypothetical protein |
| ESA_00612 | COG: Outer membrane protein and related peptidoglycan-associated (lipo)proteins |
| ESA_00613 | Hypothetical protein |
| ESA_00614 | Type I restriction-modification system methyltransferase subunit |
| ESA_00615 | Restriction endonuclease S subunits |
| ESA_00616 | Hypothetical protein |
| ESA_00617 | Type I site-specific deoxyribonuclease, HsdR family |
| ESA_00633 | Hypothetical protein |
| ESA_00756 | Hypothetical protein |
| ESA_00956 | Hypothetical protein |
| Cluster 3 |  |
| ESA_00983 | Bacterial Ig-like domain, COG5295: Autotransporter adhesin |
| ESA_00984 | Hypothetical protein |
| ESA_00985 | Hypothetical protein, COG5295 Autotransporter adhesin |
| ESA_00986 | Hypothetical protein, COG5295 Autotransporter adhesin |
| ESA_01127 | Hypothetical protein |
| ESA_01297 | Acyl-CoA synthetases (AMP-forming)/AMP-acid ligases II |
| ESA_01298 | Coenzyme F390 synthetase |
| ESA_01659 | Protein of unknown function (DUF1311). |
| ESA_01927 | Hypothetical protein |
| Cluster 4 |  |
| ESA_01935 | Cytochrome bd quinol oxidase subunit 1 apoprotein (EC 1.10.3.-) |
| ESA_01936 | Cytochrome bd quinol oxidase subunit 2 apoprotein (EC 1.10.3.-) |
| ESA_01937 | Protein of unknown function |
| ESA_01938 | Transcriptional regulator, TetR family, susc to antibiotics and detergents |
| ESA_01939 | Hypothetical protein |
| ESA_01940 | Membrane transporters of cations and cationic drugs |
| ESA_01941 | Sugar (Glycoside-Pentoside-Hexuronide) transporter |
| ESA_01942 | Alpha-L-arabinofuranosidase |
| ESA_01943 | Putative aconitase A, ZP_00835245, 65%, 7.00e-156 |
| ESA_01944 | ABC-type dipeptide transport system, periplasmic component |
| ESA_01945 | ABC-type dipeptide/oligopeptide/nickel transport systems, permease components |
| ESA_01946 | ABC-type dipeptide/oligopeptide/nickel transport systems, permease components |
| ESA_01947 | ATPase components of various ABC-type transport systems, contain duplicated ATPase |
| ESA_01948 | Coenzyme F420-dependent N5,N10-methylene tetrahydromethanopterin reductase |
| ESA_01949 | Alkylhydroperoxidase AhpD family core domain |
| ESA_01951 | Protein of unknown function |
| ESA_01953 | O-6-methylguanine DNA methyltransferase |
| ESA_01954 | Catabolite gene activator and regulatory subunit of cAMP-dependent protein kinases |
| ESA_01955 | Universal stress protein UspA and related nucleotide-binding proteins |
| ESA_01956 | NAD/NADP transhydrogenase beta subunit |
| ESA_01957 | NAD(P) transhydrogenase, alpha subunit |
| ESA_01958 | Protein of unknown function |
| ESA_01959 | Arginine/ornithine antiporter |
| ESA_01961 | Response regulators consisting of a CheY-like receiver domain and a winged-helix DNA-binding domain |
| ESA_01963 | Transcriptional regulator, LysR family |
| ESA_01964 | Signal transduction histidine kinase |
| ESA_01965 | DNA replication terminus site binding protein |
| ESA_02342 | P pilus assembly protein, pilin FimA |
| ESA_02345 | P pilus assembly protein, pilin FimA |
| Cluster 5 |  |
| ESA_02734 | Methyl-accepting chemotaxis protein, nitrate and nitrite sensing |
| ESA_02735 | Type VI secretion system lysozyme-related protein, typeVI secretion |
| ESA_02736 | Predicted virulence protein, SciE type |
| ESA_02737 | Hypothetical protein |
| ESA_02738 | Uncharacterized conserved protein |
| ESA_02753 | Holliday junction resolvase |
| ESA_02754 | Phage integrase family. |
| ESA_02832 | CRISPR-associated protein, Cas1 family |
| ESA_02833 | CRISPR-associated protein, Cse3 family |
| ESA_02834 | CRISPR-associated protein, Cas5e family |
| ESA_03042 | Hypothetical protein |
| ESA_03054 | Hypothetical protein |
| ESA_03083 | Replicative DNA helicase |
| ESA_03085 | Bacteriophage CII protein. |
| ESA_03093 | Hypothetical protein |
| ESA_03094 | cIII protein family. |
| ESA_03095 | Hypothetical protein |
| ESA_03103 | Hypothetical protein |
| ESA_03263 | Transcriptional regulator |
| ESA_03279 | Hypothetical protein |
| ESA_03436 | Hypothetical protein |
| Cluster 6 |  |
| ESA_03513 | Protein of unknown function |
| ESA_03514 | Hypothetical protein |
| ESA_03515 | P pilus assembly protein, porin PapC |
| ESA_03516 | P pilus assembly protein, chaperone PapD |
| ESA_03518 | Protein of unknown function |
| Cluster 7 |  |
| ESA_03747 | Tyrosine recombinase XerC subunit |
| ESA_03800 | TonB-dependent vitamin B12 receptor |
| ESA_03891 | Hypothetical protein |
| ESA_03892 | RHS protein |
| ESA_03929 | Hypothetical protein |
| ESA_03930 | Hypothetical protein |
| ESA_04144 | Hypothetical protein |
| ESA_04198 | Hypothetical protein |
| ESA_04305 | Protein involved in cell division |
| ESA_04306 | Hypothetical protein |
| Plasmid genes |  |
| ESA_pESA3p05433 | Hypothetical protein |
| ESA_pESA3p05435 | Putative CpmG protein involved in carbapenem resistance, NP_927554, 42%, 2.00e-28 |
| ESA_pESA3p05437 | Predicted transcriptional regulator |
| ESA_pESA3p05438 | Putative glyoxalase/bleomycin resistance protein, FuraDRAFT_0322, 45%, 2.00e-21 |
| ESA_pESA3p05439 | Mig-14, resitance to antimicrobial peptides |
| ESA_pESA3p05440 | Outer membrane protein V |
| ESA_pESA3p05442 | Signal transduction histidine kinase |
| ESA_pESA3p05443 | RND family efflux transporter, MFP subunit |
| ESA_pESA3p05444 | Cation/multidrug efflux pump, antimicroabial resistance |
| ESA_pESA3p05445 | Hypothetical protein |
| ESA_pESA3p05446 | ADP-ribose pyrophosphatase, oxidative stress protection |
| ESA_pESA3p05448 | Transcriptional regulator, tetracycline resistance |
| ESA_pESA3p05449 | ABC-type multidrug transport system, ATPase and permease componentABC transporter |
| ESA_pESA3p05450 | Metabolite-proton symporter |
| ESA_pESA3p05451 | ABC-type molybdate transport system, periplasmic component |
| ESA_pESA3p05452 | Transcriptional regulator, LysR family |
| ESA_pESA3p05453 | Hypothetical protein |
| ESA_pESA3p05454 | Transcriptional regulator, TetR family |
| ESA_pESA3p05455 | Putative antibiotic biosynthesis monooxygenase, Acid345_4767, 30%, 1.00e-09 |
| ESA_pESA3p05459 | Hypothetical protein |
| ESA_pESA3p05462 | FOG: EAL domain |
| ESA_pESA3p05463 | Predicted transcriptional regulators, mercurium resistance |
| ESA_pESA3p05464 | Methyl-accepting chemotaxis protein |
| ESA_pESA3p05465 | Putative glyoxalase/bleomycin resistance protein, BH160DRAFT_4533, 51%, 2.00e-28 |
| ESA_pESA3p05466 | N-acetylglutamate synthase and related acetyltransferases |
| ESA_pESA3p05467 | Hypothetical protein |
| ESA_pESA3p05468 | Uncharacterized enzyme involved in biosynthesis of extracellular polysaccharides |
| ESA_pESA3p05469 | ACT domain-containing protein |
| ESA_pESA3p05470 | Predicted transcriptional regulators |
| ESA_pESA3p05471 | Predicted hydrolases or acyltransferases (alpha/beta hydrolase superfamily) |
| ESA_pESA3p05472 | PTS system IIB component/PTS system IIC component |
| ESA_pESA3p05473 | L-alanine-DL-glutamate epimerase and related enzymes of enolase superfamily |
| ESA_pESA3p05474 | Transcriptional regulator, GntR family |
| ESA_pESA3p05475 | Predicted membrane protein |
| ESA_pESA3p05476 | Hypothetical protein |
| ESA_pESA3p05478 | Diguanylate cyclase (GGDEF) domain |
| ESA_pESA3p05479 | Hypothetical protein |
| ESA_pESA3p05482 | Hypothetical protein |
| ESA_pESA3p05484 | Potassium uptake protein, TrkH family |
| ESA_pESA3p05485 | Arsenate reductase (glutaredoxin) |
| ESA_pESA3p05490 | Transcriptional regulator, GntR family |
| ESA_pESA3p05513 | SMI1 / KNR4 family. |

^1^ Names are those attributed by JGI-IMG (http://img.jgi.doe.gov/). Where a product name was attributed using a Blast homology search, the accession number, amino acid percent identity and E-value are listed.
