# Supplementary material for: Genome Sequence of Cronobacter sakazakii BAA-894 and Comparative Genomic Hybridization Analysis with Other Cronobacter Species
Source: PLoS One. 2010 Mar 8;5(3):e9556. doi: 10.1371/journal.pone.0009556 (PMC2833190; doi:10.1371/journal.pone.0009556)
Supplement: Table S2 — Non-phage genomic regions absent in more than half of the tested strains. (0.40 MB DOC) [file pone.0009556.s003.doc]

**Table S2.** Non-phage genomic regions absent in more than half of the tested strains.

| Gene locus | Product description |
| --- | --- |
| GR1 |  |
| ESA_00140 | Hypothetical protein |
| ESA_00141 | Uncharacterized protein conserved in bacteria |
| ESA_00142 | type VI secretion system, Vgr family protein |
| ESA_00143 | type VI secretion protein, VC_A0110 family |
| ESA_00144 | type VI secretion protein, VC_A0111 family |
| ESA_00145 | type VI secretion lipoprotein, VC_A0113 family |
| GR2 |  |
| ESA_00292 | Rhs element Vgr protein |
| ESA_00293 | Hypothetical protein |
| ESA_00294 | Hypothetical protein |
| ESA_00296 | Hypothetical protein |
| ESA_00297 | Hypothetical protein |
| ESA_00298 | VRR-NUC domain. |
| ESA_00299 | Hypothetical protein, family: beta lactamases A (IPR000871) |
| ESA_00300 | Hypothetical protein |
| ESA_00301 | Hypothetical protein |
| ESA_00302 | Hypothetical protein |
| ESA_00303 | Predicted membrane protein involved in D-alanine export |
| ESA_00304 | Uncharacterized protein conserved in bacteria |
| ESA_00305 | Hypothetical protein |
| ESA_00306 | Uncharacterized protein conserved in bacteria |
| ESA_00307 | Uncharacterized protein conserved in bacteria |
| ESA_00308 | Large extracellular alpha-helical protein |
| ESA_00309 | Predicted secreted protein |
| ESA_00310 | Uncharacterized protein conserved in bacteria |
| GR3, putative prophage fragment 1 | |
| ESA_00604 | Hypothetical protein |
| ESA_00605 | Hypothetical protein |
| ESA_00606 | Hypothetical protein |
| ESA_00607 | Protein of unknown function |
| ESA_00609 | Hypothetical protein |
| ESA_00610 | Superfamily II DNA/RNA helicases, SNF2 family |
| ESA_00611 | Hypothetical protein |
| ESA_00612 | COG: Outer membrane protein and related peptidoglycan-associated (lipo)proteins |
| ESA_00613 | Ferritin related (IPR) |
| ESA_00614 | Type I restriction-modification system methyltransferase subunit |
| ESA_00615 | Restriction endonuclease S subunits |
| ESA_00616 | Hypothetical protein |
| ESA_00617 | Type I site-specific deoxyribonuclease, HsdR family |
| ESA_00618 | Putative EA59 phage protein, RPA2566, 46%, 4.00e-120 |
| ESA_00619 | Hypothetical protein |
| ESA_00620 | Plasmid and phage DNA primase |
| ESA_00621 | Hypothetical protein |
| ESA_00622 | P4 phage protein |
| ESA_00624 | Ash protein family (phage) |
| ESA_00625 | Phage transcriptional regulator, AlpA |
| ESA_00626 | Putative phage capsid protein |
| ESA_00627 | Phage transcriptional activator, Ogr/Delta |
| ESA_00628 | Hypothetical protein |
| ESA_00629 | Hypothetical protein |
| ESA_00630 | Phage integrase |
| GR4, putative prophage 1 | |
| ESA_00990 | Putative phage integrase |
| ESA_00991 | Hypothetical protein |
| ESA_00992 | Putative phage exonuclease |
| ESA_00993 | Hypothetical protein |
| ESA_00994 | Hypothetical protein |
| ESA_00995 | Putative methyltransferase |
| ESA_00996 | Hypothetical protein |
| ESA_00997 | Eae-like protein, YP_002225111, 40%, 2.00e-83 |
| ESA_00998 | Recombination associated protein RdgC |
| ESA_00999 | Hypothetical protein |
| ESA_01000 | Hypothetical protein |
| ESA_01001 | NAD-dependent DNA ligase |
| ESA_01002 | Putative phage repressor |
| ESA_01003 | Lambda repressor - like protein |
| ESA_01004 | Putative nucleic acid-binding protein, YP_002381770, 63%, 3.00e-62 |
| ESA_01005 | Hypothetical protein |
| ESA_01006 | Phage terminase-like protein, large subunit |
| ESA_01007 | Hypothetical protein |
| ESA_01008 | Conserved phage C-terminus (Phg_2220_C) |
| ESA_01009 | Hypothetical protein |
| ESA_01010 | DNA adenine methylase |
| ESA_01011 | SOS-response transcriptional repressors (lexA) |
| ESA_01012 | Endodeoxyribonuclease RusA |
| ESA_01013 | Phage antirepressor, ZP_03842144, 59, 2.00e-67 |
| ESA_01014 | Hypothetical protein |
| ESA_01015 | Antitermination protein |
| ESA_01016 | Hypothetical protein |
| ESA_01017 | Putative phage membrane protein, YP_001907872, 61%, 3.00e-32 |
| ESA_01018 | Putative phage membrane protein, YP_0011907871, 69%, 7.00e-30 |
| ESA_01019 | Phage-encoded lysozyme, YP_002244041, 67%, 30.00e-70 |
| ESA_01020 | Bacteriophage lysis protein. |
| ESA_01021 | Hypothetical protein |
| ESA_01022 | Hypothetical protein |
| ESA_01023 | Hypothetical protein |
| ESA_01024 | Phage terminase, small subunit, putative, P27 family |
| ESA_01025 | Phage terminase-like protein, large subunit |
| ESA_01026 | Phage portal protein, HK97 family |
| ESA_01027 | Protease subunit of ATP-dependent Clp proteases |
| ESA_01028 | Phage major capsid protein, HK97 family |
| ESA_01029 | Hypothetical protein |
| ESA_01030 | Uncharacterized phage protein, ZP_02661970, 49%, 9.00e-22 |
| ESA_01031 | Phage head-tail adaptor, putative, SPP1 family |
| ESA_01032 | Phage tail protein, HK97 gp10 family |
| ESA_01033 | Hypothetical protein |
| ESA_01034 | Major phage tail subunit, NP_037706, 58%, 1.00e-41 |
| ESA_01035 | Phage tail assembly chaperone |
| ESA_01036 | Putative phage tail protein, ZP_03085521, 51%, 8.00e-17 |
| ESA_01037 | Hypothetical protein |
| ESA_01038 | Phage-related membrane protein, ZP_02500840, 39%, 6.00e-09 |
| ESA_01039 | Tail tape measure protein |
| ESA_01040 | Phage-related protein (minor tail protein) |
| ESA_01041 | Lambda-like phage minor tail protein L |
| ESA_01042 | Tail assembly protein |
| ESA_01043 | Lambda-like tail assembly protein |
| ESA_01044 | Phage-related protein, tail component |
| ESA_01045 | Hypothetical protein |
| ESA_01046 | Tail fiber assembly protein |
| ESA_01047 | tRNA-Pro-GGG |
| ESA_01048 | Predicted sulfatase |
| ESA_01049 | Hypothetical protein |
| ESA_01050 | Phage head morphogenesis protein, SPP1 gp7 family |
| ESA_01051 | LSU ribosomal protein L25P |
| ESA_01052 | DNA or RNA helicases of superfamily II |
| GR5 |  |
| ESA_01179 | Glucose-1-phosphate thymidylyltransferase (EC 2.7.7.24) |
| ESA_01180 | Acetyltransferases, including N-acetylases of ribosomal proteins |
| ESA_01181 | Hypothetical protein |
| ESA_01182 | Predicted pyridoxal phosphate-dependent enzyme,regulation of cell wall biogenesis |
| ESA_01183 | Membrane protein involved in the export of O-antigen and teichoic acid |
| ESA_01184 | Predicted glycosyltransferases |
| ESA_01185 | Hypothetical protein |
| ESA_01186 | Hypothetical protein |
| ESA_01187 | Hypothetical protein |
| ESA_01188 | Glycosyltransferases involved in cell wall biogenesis |
| ESA_01189 | 6-phosphogluconate dehydrogenase (decarboxylating) (EC 1.1.1.44) |
| GR6, putative prophage 2 | |
| ESA_01608 | Phage integrase, lambda-like |
| ESA_01609 | Putative phage excisionase |
| ESA_01610 | Putative bacteriophage protein, YP_689300, 84%, 4.00e-182 |
| ESA_01611 | Host-nuclease inhibitor protein Gam |
| ESA_01612 | Putative phage repressor, lambda-like |
| ESA_01613 | Repressor CII protein, lambda-like |
| ESA_01614 | Bacteriophage replication protein O |
| ESA_01615 | Phage replication protein P, lambda-like |
| ESA_01616 | Predictred ATPase |
| ESA_01617 | Hypothetical protein |
| ESA_01618 | Conserved phage protein, YP_002411362, 68%, 2.00e-60 |
| ESA_01619 | Hypothetical protein |
| ESA_01620 | Hypothetical protein |
| ESA_01621 | Endodeoxiribonuclease RusA |
| ESA_01622 | Phage antitermination protein Q. |
| ESA_01623 | Hypothetical protein |
| ESA_01624 | S-lysis protein, Qin phage |
| ESA_01625 | Phage-related lysozyme (muraminidase) |
| ESA_01626 | Hypothetical protein |
| ESA_01627 | Hypothetical protein |
| ESA_01628 | Sigma S-stabilisation anti-adaptor protein |
| ESA_01629 | Hypothetical protein |
| ESA_01630 | Hypothetical protein |
| ESA_01631 | Hypothetical protein |
| ESA_01632 | Putative phage terminase, small subunit, P27 family |
| ESA_01633 | Phage terminase, large subunit |
| ESA_01634 | Hypothetical protein |
| ESA_01635 | Phage portal protein, HK97 family |
| ESA_01636 | Phage prohead protease, HK97 family |
| ESA_01637 | Phage major capsid protein, HK97 family |
| ESA_01638 | Phage tail protein, Ig-like (bacterial surface) |
| ESA_01639 | Uncharacterized phage protein, YP_001177316, 60%, 8.00e-31 |
| ESA_01640 | Putative phage head-tail adaptor, SPP1 family |
| ESA_01641 | Putative phage protein, YP_687824, 70%, 1.00e-63 |
| ESA_01642 | Hypothetical protein, |
| ESA_01643 | Phage tail protein, YP_001879678, 52%, 6.00e-52 |
| ESA_01644 | Putative phage tail fiber protein, YP_002117170, 29%, 4.00e-27 |
| GR7 |  |
| ESA_01775 | Protein of unknown function |
| ESA_01776 | Hypothetical protein |
| ESA_01777 | Hypothetical protein |
| ESA_01778 | Transposase and inactivated derivatives |
| ESA_01779 | Hypothetical protein |
| ESA_01780 | Putative transposase, Ent638_0739, 95%, 6.00e-04 |
| ESA_01781 | Hypothetical protein |
| ESA_01782 | Hypothetical protein |
| ESA_01783 | Uncharacterized protein encoded in toxicity protection region of plasmid R478 |
| ESA_01784 | Uncharacterized protein encoded in toxicity protection region of plasmid R478 |
| ESA_01785 | Uncharacterized proteins involved in stress response, homologs of TerZ |
| ESA_01786 | Uncharacterized protein encoded in toxicity protection region of plasmid R478 |
| ESA_01787 | Hypothetical protein |
| ESA_01788 | Hypothetical protein |
| ESA_01789 | Tellurite resistance protein TerW, APECO1_O1R61, 99%, 1.00e-78 |
| ESA_01790 | Hypothetical protein |
| ESA_01791 | Citrate lyase beta subunit |
| ESA_01792 | Predicted nucleoside-diphosphate sugar epimerases, EcolE1_01003643, 97%, 0.00e+00 |
| ESA_01793 | Hypothetical protein |
| ESA_01794 | Hypothetical protein |
| ESA_01795 | ATP-grasp domain. |
| ESA_01796 | Uncharacterized proteins involved in stress response, homologs of TerZ , TerA |
| ESA_01797 | Uncharacterized proteins involved in stress response, homologs of TerZ ,TerB |
| ESA_01798 | Tellurite resistance protein |
| ESA_01799 | Membrane protein TerC, possibly involved in tellurium resistance |
| ESA_01800 | Uncharacterized proteins involved in stress response, homologs of Ter, TerD |
| ESA_01801 | Uncharacterized proteins involved in stress response, homologs of Ter, TerD |
| ESA_01802 | Uncharacterized proteins involved in stress response, homologs of Ter, TerD |
| ESA_01803 | Putative transposase, APECO1_345, 71%, 5.00e-27 |
| ESA_01804 | Hypothetical protein |
| GR8 |  |
| ESA_01970 | P pilus assembly protein, pilin FimA |
| ESA_01971 | P pilus assembly protein, pilin FimA |
| ESA_01972 | P pilus assembly protein, pilin FimA |
| ESA_01973 | P pilus assembly protein, pilin FimA |
| ESA_01974 | P pilus assembly protein, porin PapC |
| ESA_01975 | P pilus assembly protein, chaperone PapD |
| ESA_01976 | P pilus assembly protein, pilin FimA |
| GR9 |  |
| ESA_02032 | Hypothetical protein |
| ESA_02033 | Hypothetical protein |
| ESA_02034 | Hypothetical protein |
| ESA_02035 | Uncharacterized protein conserved in bacteria |
| ESA_02036 | Uncharacterized protein conserved in bacteria |
| ESA_02037 | type VI secretion system Vgr family protein |
| ESA_02038 | type VI secretion lipoprotein, VC_A0113 family |
| ESA_02039 | type VI secretion protein, VC_A0111 family |
| ESA_02040 | type VI secretion protein, VC_A0110 family |
| ESA_02041 | Uncharacterized protein conserved in bacteria |
| GR10, putative prophage fragment 2 |  |
| ESA_02304 | Hypothetical protein |
| ESA_02305 | Hypothetical protein |
| ESA_02306 | Hypothetical protein |
| ESA_02307 | Hypothetical protein |
| ESA_02308 | Hypothetical protein |
| ESA_02309 | Phage lysozyme |
| ESA_02310 | Hypothetical protein |
| ESA_02311 | Fibronectin type III domain, COG: phage tail component |
| ESA_02312 | Hypothetical protein |
| ESA_02313 | Putative phage tape measure protein, PAJU2_gp18, 20%, 1.00e-13 |
| ESA_02314 | Hypothetical protein |
| ESA_02315 | Hypothetical protein |
| ESA_02316 | Putative phage protein, Ea21-4_gp72, 36%, 1.00e-125 |
| ESA_02317 | Hypothetical protein |
| ESA_02318 | Hypothetical protein |
| ESA_02319 | Putative phage protein, PcarcW_010200019589, 25%, 9.00e-23 |
| ESA_02320 | Phage portal protein, lambda family |
| ESA_02321 | Hypothetical protein |
| ESA_02322 | Hypothetical protein |
| ESA_02323 | Hypothetical protein |
| ESA_02324 | Hypothetical protein |
| ESA_02325 | Hypothetical protein |
| ESA_02326 | Hypothetical protein |
| ESA_02327 | EAL domain |
| ESA_02328 | Hypothetical protein |
| ESA_02329 | Hypothetical protein |
| ESA_02330 | Hypothetical protein |
| ESA_02331 | Hypothetical protein |
| ESA_02332 | Hypothetical protein |
| ESA_02334 | Hypothetical protein |
| ESA_02335 | Hypothetical protein |
| ESA_02336 | Hypothetical protein |
| ESA_02337 | Hypothetical protein |
| ESA_02338 | Hypothetical protein |
| ESA_02339 | Site-specific recombinases, DNA invertase Pin homologs |
| GR11, putative prophage fragment 3 | |
| ESA_02740 | Membrane protein involved in colicin uptake, YP_002390565, 30%, 7.00e-04 |
| ESA_02741 | Phage head morphogenesis protein, SPP1 gp7 family |
| ESA_02742 | Hypothetical protein |
| ESA_02743 | Phage terminase, large subunit, YP_002113600, 97%, 3.00e-70 |
| ESA_02744 | Phage terminase, small subunit, NP_112075, 77%, 4.00e-49 |
| ESA_02745 | Hypothetical protein |
| ESA_02746 | Bacteriophage lysis protein |
| ESA_02747 | Phage-related lysozyme (muraminidase) |
| ESA_02748 | Lysis protein S |
| ESA_02749 | Hypothetical protein |
| ESA_02750 | Antitermination protein |
| ESA_02751 | NinZ - phage protein (similar to P22), NP_720317, 42%, 8.00e-04 |
| ESA_02752 | Hypothetical protein |
| ESA_02753 | Endodeoxyribonuclease RusA |
| ESA_02754 | Phage integrase |
| ESA_02755 | tRNA-Arg-TCT |
| GR12, putative prophage 3 |  |
| ESA_03025 | Integrase |
| ESA_03026 | O antigen conversion: glucose translocase, NP_599057, 79%, 3.00e-50 |
| ESA_03027 | Phage bactoprenol glucosyl transferase, NP_416852, 90%, 5.00e-157 |
| ESA_03028 | O antigen conversion: translocase, YP_151580, 27%, 2.00e-30 |
| ESA_03029 | Phage T7 tail fibre protein. |
| ESA_03030 | Putative phage tail component protein |
| ESA_03031 | Tail assembly protein, YP_001742074, 36%, 1.00e-22 |
| ESA_03032 | Hypothetical protein |
| ESA_03033 | Hypothetical protein |
| ESA_03034 | Phage tape measure protein |
| ESA_03035 | Hypothetical protein |
| ESA_03036 | Hypothetical protein |
| ESA_03037 | Hypothetical protein |
| ESA_03038 | Predicted glycosyltransferase, YP_002225111, 40%, 2.00e-83 |
| ESA_03039 | Major phage tail subunit, YP_001742066, 49%, 6.00e-37 |
| ESA_03040 | Hypothetical protein |
| ESA_03041 | Hypothetical protein |
| ESA_03042 | Hypothetical protein |
| ESA_03043 | Hypothetical protein |
| ESA_03044 | Hypothetical protein |
| ESA_03045 | Hypothetical protein |
| ESA_03046 | Hypothetical protein |
| ESA_03047 | Putative capsid decoration protein |
| ESA_03048 | Putative colicin uptake protein TolA, YP_539759, 30%, 8.00e-04 |
| ESA_03049 | Hypothetical protein |
| ESA_03050 | Phage head morphogenesis protein, SPP1 gp7 family |
| ESA_03051 | Putative phage portal protein, YP_001742041, 40%, 2.00e-94 |
| ESA_03052 | Phage terminase - large subunit |
| ESA_03053 | Phage terminase - small subunit |
| ESA_03054 | Hypothetical protein |
| ESA_03055 | Hypothetical protein |
| ESA_03056 | Hypothetical protein |
| ESA_03057 | Hypothetical protein |
| ESA_03058 | Phage regulatory protein, possible antirepressor |
| ESA_03059 | Hypothetical protein |
| ESA_03060 | Hypothetical protein |
| ESA_03061 | Hypothetical protein |
| ESA_03062 | Phage-related lysozyme (muraminidase) |
| ESA_03063 | Hypothetical protein |
| ESA_03064 | Hypothetical protein |
| ESA_03065 | Hypothetical protein |
| ESA_03066 | Hypothetical protein |
| ESA_03067 | Hypothetical protein |
| ESA_03068 | Phage protein (similar to NinZ), NP_720317, 42%, 8.00e-04 |
| ESA_03069 | Hypothetical protein |
| ESA_03070 | Bacteriophage Lambda NinG protein |
| ESA_03071 | Hypothetical protein |
| ESA_03072 | NinF protein |
| ESA_03073 | Hypothetical protein |
| ESA_03074 | Hypothetical protein |
| ESA_03075 | NinB protein |
| ESA_03076 | Phage protein ASCH domain |
| ESA_03077 | Phage protein, YP_002383185, 41%, 3.00e-08 |
| ESA_03078 | Hypothetical protein |
| ESA_03079 | Hypothetical protein |
| ESA_03080 | Hypothetical protein |
| ESA_03081 | Hypothetical protein |
| ESA_03052 | Phage terminase - large subunit |
| ESA_03053 | Phage terminase - small subunit |
| ESA_03054 | Hypothetical protein |
| ESA_03055 | Hypothetical protein |
| ESA_03056 | Hypothetical protein |
| ESA_03057 | Hypothetical protein |
| ESA_03058 | Phage regulatory protein, possible antirepressor |
| ESA_03059 | Hypothetical protein |
| ESA_03060 | Hypothetical protein |
| ESA_03082 | Phage protein, NP_597892, 65%, 1.00e-27 |
| ESA_03083 | Replication protein P |
| ESA_03084 | Replication protein O, ZP_02707084, 68%, 9.00e-11 |
| ESA_03085 | Bacteriophage CII protein. |
| ESA_03086 | Phage repressor protein (CI) |
| ESA_03087 | Hypothetical protein |
| ESA_03088 | Predicted early gene regulator, YP_002328239, 61%, 1.00e-16 |
| ESA_03089 | Hypothetical protein |
| ESA_03090 | Hypothetical protein |
| ESA_03091 | Hypothetical protein |
| ESA_03092 | Phage protein, YP_0017442057, 45%, 2.00e-07 |
| ESA_03093 | Hypothetical protein |
| ESA_03094 | CIII protein family |
| ESA_03095 | Lambda bacteriophage Kil protein, YP_541680, 67%. 1.00e-11 |
| ESA_03096 | Hypothetical protein |
| ESA_03097 | Phage protein, NP_112046, 48%, 1.00e-04 |
| ESA_03098 | ERF superfamily |
| ESA_03099 | Hypothetical protein |
| ESA_03100 | Hypothetical protein |
| ESA_03101 | Phage protein, NP_037723, 78%, 2.00e-06 |
| ESA_03102 | Hypothetical protein |
| GR13 |  |
| ESA_03887 | SMI1 / KNR4 family (cell wall formation) |
| ESA_03888 | hypothetical protein |
| ESA_03889 | hypothetical protein |
| ESA_03890 | hypothetical protein |
| ESA_03893 | hypothetical protein |
| ESA_03894 | hypothetical protein |
| ESA_03895 | hypothetical protein |
| ESA_03896 | hypothetical protein |
| ESA_03897 | hypothetical protein |
| ESA_03898 | hypothetical protein |
| ESA_03900 | hypothetical protein |
| ESA_03902 | hypothetical protein |
| ESA_03911 | hypothetical protein |
| ESA_03912 | hypothetical protein |
| GR14 |  |
| ESA_04248 | Probable copper-binding protein, YP_001965802, 45%, 2.0e-12 |
| ESA_04249 | Copper-resistance protein, CopA family |
| ESA_04250 | Uncharacterized protein involved in copper resistance |
| ESA_04251 | Uncharacterized protein, homolog of Cu resistance protein CopC |
| ESA_04252 | Putative copper export protein |
| ESA_04253 | Heavy metal response regulator |
| ESA_04254 | Heavy metal sensor kinase |
| ESA_04255 | Probable copper binding protein, SMR0159, 100%, 2.00e-75 |
| GR15 |  |
| ESA_pESA3p05493 | Type VI secretion protein, VC_A0114 family |
| ESA_pESA3p05494 | Type IV / VI secretion system protein, DotU family |
| ESA_pESA3p05495 | Outer membrane protein and related peptidoglycan-associated (lipo)proteins, OmpA family |
| ESA_pESA3p05497 | Type VI secretion ATPase, ClpV1 family |
| ESA_pESA3p05498 | Hypothetical protein |
| ESA_pESA3p05499 | Hypothetical protein |
| ESA_pESA3p05500 | Type VI secretion system Vgr family protein |
| ESA_pESA3p05503 | Uncharacterized protein conserved in bacteria |
| ESA_pESA3p05504 | Uncharacterized protein conserved in bacteria |
| ESA_pESA3p05505 | Hypothetical protein |

^1^ Names are those attributed by JGI-IMG (http://img.jgi.doe.gov/). Where a product name was attributed using a Blast homology search, the accession number, amino acid percent identity and E-value are listed.
