# Supplementary material for: Genome Sequence of Cronobacter sakazakii BAA-894 and Comparative Genomic Hybridization Analysis with Other Cronobacter Species
Source: PLoS One. 2010 Mar 8;5(3):e9556. doi: 10.1371/journal.pone.0009556 (PMC2833190; doi:10.1371/journal.pone.0009556)
Supplement: Table S3 — Cut-off values used for the assignment of absent, intermediate or present gene status. (0.04 MB DOC) [file pone.0009556.s004.doc]

**Table S3.** Cut-off values used for the assignment of absent, intermediate or present gene status.

| Organism | absent / intermediate | intermediate / present |
| --- | --- | --- |
| *C. sakazakii* ATCC 29544^T^ | 0.51 | 0.93 |
| *C. sakazakii* ATCC 12868 | 0.54 | 1.03 |
| *C. sakazakii* strain 20 | 0.45 | 0.85 |
| *C. sakazakii* strain 701 | 0.62 | 1.01 |
| *C. sakazakii* strain 767 | 0.66 | 1.05 |
| *C. sakazakii* strain 696 | 0.46 | 0.89 |
| *C. malonaticus* LMG 23826^T^ | 0.16 | 0.57 |
| *C. turicensis* LMG 23827^T^ | 0.11 | 0.43 |
| *C. muytjensiii* ATCC 51329^T^ | 0.10 | 0.31 |
| *C. dublinensis* LMG 23823^T^ | 0.11 | 0.32 |
